# Established Microbial Colonies Can Survive Type VI Secretion Assault

**DOI:** 10.1371/journal.pcbi.1004520

**Published:** 2015-10-20

**Authors:** David Bruce Borenstein, Peter Ringel, Marek Basler, Ned S. Wingreen

**Affiliations:** 1 Princeton University, Lewis-Sigler Institute for Integrative Genomics, Princeton, New Jersey, United States of America; 2 Universität Basel, Biozentrum, Basel, Switzerland; 3 Princeton University, Department of Molecular Biology, Princeton, New Jersey, United States of America; ETH Zurich, SWITZERLAND

## Abstract

Type VI secretion (T6S) is a cell-to-cell injection system that can be used as a microbial weapon. T6S kills vulnerable cells, and is present in close to 25% of sequenced Gram-negative bacteria. To examine the ecological role of T6S among bacteria, we competed self-immune T6S+ cells and T6S-sensitive cells in simulated range expansions. As killing takes place only at the interface between sensitive and T6S+ strains, while growth takes place everywhere, sufficiently large domains of sensitive cells can achieve net growth in the face of attack. Indeed T6S-sensitive cells can often outgrow their T6S+ competitors. We validated these findings through *in vivo* competition experiments between T6S+ *Vibrio cholerae* and T6S-sensitive *Escherichia coli*. We found that *E. coli* can survive and even dominate so long as they have an adequate opportunity to form microcolonies at the outset of the competition. Finally, in simulated competitions between two equivalent and mutually sensitive T6S+ strains, the more numerous strain has an advantage that increases with the T6S attack rate. We conclude that sufficiently large domains of T6S-sensitive individuals can survive attack and potentially outcompete self-immune T6S+ bacteria.

## Author Summary

Type VI secretion (T6S) is a cell-to-cell injection system that can be used as a microbial weapon. T6S kills vulnerable cells, and is present in a significant fraction of bacteria. Given the tactical advantage conferred by T6S, the system’s lack of universality suggests limits to its effectiveness relative to its costs. In our study, we use theory and experiments to identify the limits of T6S as a cell-to-cell weapon. We find that cell birth inside an existing colony can offset cell death due to T6S killing at the colony’s edge, helping sufficiently large (“established”) groups of sensitive cells to survive. T6S has been extensively studied because of its implications in both disease and inter-microbial competition. The present study is the first to identify the practical limits of T6S as a killing mechanism.

## Introduction

Microbes employ a staggering range of extracellular tools to engineer their immediate environment [1–6]. Very often, that environment is defined by the multitude of other cells in close proximity. These neighbors pose both a threat and an opportunity, and represent an important target for manipulation [7–10].

**Fig 1:**
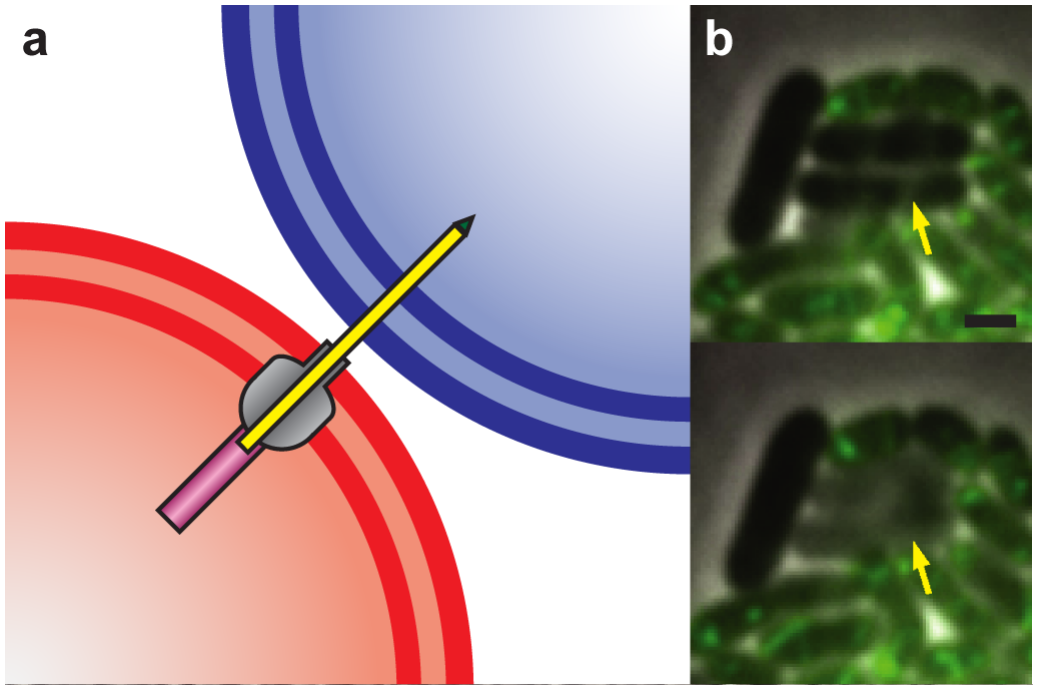
Function and mechanism of the T6S system. (a) The T6S system consists of a contractile outer sheath (purple), an inner tube (yellow), a membrane complex and baseplate (grey) and spike proteins (green). The contractile sheath pushes the inner tube through the baseplate and membrane complex, causing the tube to penetrate the target cell. (b) Competition between *V. cholerae* str. 2740–80 (sheath labeled with GFP) and *E. coli* MG1655 (unlabeled). Arrow shows *E. coli* cells that undergo lysis. Panels are taken two minutes apart; scale bar 1 *μ*m.

The Type VI secretion system (T6SS) is a mechanism for direct cell-to-cell manipulation through the translocation of effector proteins. The T6SS consists of a helical sheath, surrounding an inner tube with associated effectors, and a baseplate attached to the bacterial cell wall (Fig 1a) [11, 12]. The T6SS is functionally close to the contractile phage tail, with which it shares evolutionary origins [13–17]. When triggered, the sheath contracts rapidly, pushing the effector through a specialized pore and into a neighboring cell [18–22].

Specialized T6SSs can directly damage both prokaryotic and eukaryotic target cells through the translocation of toxic proteins directly into the target cell. T6SSs are observed to cause death via numerous mechanisms in both bacteria and eukaryotes (Fig 1b; S1 Video) [13, 18, 23–28]. In fact, many species have developed multiple, specialized T6SSs [26]; for example, *Burkholderia thailandensis* has five separate T6SSs, which allow it to attack both prokaryotic and eukaryotic cells [29]. T6SSs are present in approximately 25% of the Gram-negative genomes studied by Boyer and colleagues [30]. Antibacterial T6SSs appear to be found with cognate immunity proteins in every case [26]. Given this tactical advantage, one might expect T6S to be even more widespread. The lack of universality of the T6SS suggests that there are limits to its utility relative to its costs.

To address the question of T6S’s utility, we focused on the case of cell-to-cell killing between bacteria. We explored this scenario through the use of individual-based models (IBMs; also called “agent-based models”). IBMs simulate the behavior of many, possibly different individuals each of which obeys rules that dictate the individual’s behavior as a function of its immediate environment. IBMs are a common tool in ecology, and have been widely used in the study of spatially explicit biological processes. Examples at the multicellular scale include the evolution of cancer, the spread of disease, and the dispersal of plants [31–38]; IBMs are also used to study dynamics at the subcellular scale [39]. More generally, IBMs have been used to address a wide range of questions concerning cooperation and conflict, of which T6S strategy can be viewed as an example [40–45].

In this study, we develop a series of IBMs. The first competes self-immune T6S+ and sensitive individuals in a range expansion, analogous to a surface colony (2D) or a biofilm (3D). We find that cell growth from the inside of a sufficiently large (or “established”) domain can offset cell death at the interface between a T6S-sensitive strain and a self-immune T6S attacker. Consequently, given a sufficiently large domain, T6S-sensitive strains can survive T6S attack. The sensitive strain does not require a growth advantage to survive; in fact, the sensitive strain can resist elimination even with a slower growth rate. Given even a small growth advantage, the T6S-sensitive strain can outcompete a self-immune T6S+ competitor. In a variant on the original model, we also find that moderate nutrient limitation has a negligible effect on competition outcomes.

We validated these findings through *in vivo* competition experiments between T6S+ *Vibrio cholerae* and T6S-sensitive *Escherichia coli*. In these 2D plate assays, *E. coli* can form microcolonies that survive, provided the initial local density of *V. cholerae* is not too high. Along similar lines, simulated competitions between mutually sensitive T6S+ strains (strains that are self-immune but sensitive to one another) reveal that the initially more numerous strain benefits most from higher attack rates. We conclude with a discussion of the ecological impact of T6SSs.

## Materials and Methods

### Competition experiments

*Escherichia coli* MG1655 Gent^R^ (LacZ+) was competed against *Vibrio cholerae* str. 2740–80 (LacZ-), similarly to what was described previously [19]. *E. coli* and *V. cholerae* were each grown from frozen stocks in Luria-Bertani broth (LB), supplemented with the appropriate antibiotic, shaking overnight at 37°C and 200 rpm. The cells were washed twice with LB before being diluted to an OD_600nm_ of 0.5. To confirm that the initial number of viable cells were comparable among the competition assays, the colony forming units (CFUs) were determined by serially diluting the washed and diluted *V. cholerae* and *E. coli* cultures 10-fold in 96-well plates in triplicate. Thereafter, 5 *μ*L of each dilution were spotted on an LB agar plate (LA).

For the competition assays, the cultures were mixed in a 1:1 ratio, which was then serially diluted 3-fold in a 96-well plate. For selected dilutions 5 *μ*L were spotted on a LA/IPTG 100 *μ*M/X-Gal 40 *μ*g/mL plate in duplicate. The competition plates were incubated at 37°C overnight. To determine the *E. coli* to *V. cholerae* ratios resulting from the competition assays, the CFUs of both strains were determined for each spot. This was achieved by excising the spots from the competition assay plates and resuspendig the bacteria in 1 mL LB by vigorously vortexing for at least 15 sec. These suspensions were serially diluted 10-fold in 96-well plates and 5 *μ*L of each dilution were spotted on LA plates supplemented with the appropriate antibiotic. The CFU plates where either incubated at 37°C overnight or at lower temperatures until colonies were visible. Images of the plates were taken on a white light transilluminator. Timelapse movies of the competition assay were obtained by preparing the competition assay plates and the pre-competition CFU plates as described before, except that the competition mixtures were only spotted once. The competition assay plate was incubated at 37°C on a white light transilluminator while taking an image every 10 min over 24 h using a Nikon D5200. The contrast, brightness and white balance of the images were adjusted using Adobe Photoshop CS5. The same settings were applied to all timelapse images. Thereafter the images were further processed and converted to a video using Fiji [46].

The growth rate determination was carried out under the same conditions as the killing assay. The same cultures (OD_600nm_ = 0.5) were individually spotted on LA plates and incubated at 37°C. Every hour the CFU was determined from a spot of each strain, as described for the endpoint killing assay. The growth rate was then derived from the parameters of the fit of an exponential curve. For the *E. coli* MG1655 Gent^R^ overnight cultures and selective CFU plates the growth medium was supplemented with 15 *μ*g /mL Gentamicin, whereas for *V. cholerae* str. 2740–80 50 *μ*g /mL Streptomycin was added.

Imaging of a competition between *E. coli* and *V. cholerae* VipA-msfGFP strains was performed under conditions similar to those used previously for imaging of T6SS activity in *V. cholerae* [17]. Strains were grown to OD_600nm_ ≈ 1 and mixed at a 1:1 ratio on an LB 1% agarose pad. Imaging started after 10–20 min and was performed at 37°C for the indicated number of frames and at the indicated frame rate.

### Simulations

Computer models were implemented using Nanoverse 0.x, a prototype of our freely available individual-based modeling platform [47]. In Nanoverse, individual agents (e.g. cells) occupy spaces on a regular lattice. In every step of a simulation, one or more individuals perform a series of behaviors; if multiple individuals act simultaneously, the events are resolved in random order.

**Fig 2:**
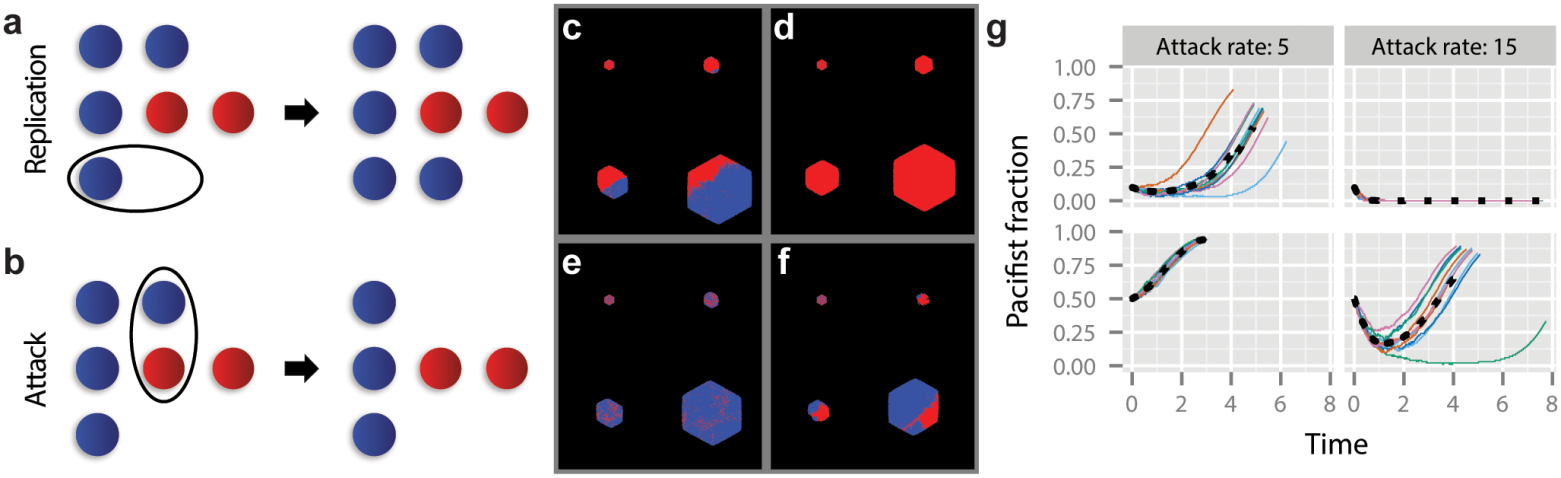
A simple spatial model of T6S-driven community dynamics. (a) Any cell can divide. Division results in an identical cell being placed in an adjacent site. If no adjacent site is available, cells are pushed out of the way to make room for the new cell (S1 Text). (b) T6S+ cells (red) can attack any cell. When a sensitive cell (blue) is attacked, it is “killed” (removed from the system). T6S+ strains are self-immune. (c-f) Time series of competitions between a T6S+ strain (red) and a sensitive strain (blue) during a range expansion in 2D. In all cases the T6S+ growth rate is *α*_*t*_ = 1, and the sensitive strain growth rate is *α*_*s*_ = 4. Initial sensitive strain fractions are 0.1 (c, d) and 0.5 (e, f). Attack rates are *γ* = 5 (c, e) and 15 (d, f). (g) Quantification of dynamics observed in panels (c-f). Thin colored lines are individual trajectories; dotted black lines are averages over 8 of the 10 cases shown (eliminating highest and lowest outliers). Parameters are as in the time series. Time is units of 1/*α*_*t*_. Timestep multiplier *λ* = 1.

Two types of individual cells are included in the simulations (Fig 2a and 2b): self-immune T6S+ (“T6S+”) cells, shown in red, and sensitive T6S- (“sensitive”) cells, shown in blue. (Self-sensitive T6S+ strains “self-destruct” rapidly in simulations, and indeed have not been observed in nature.) Every cell has an associated probability of cell division per step of the simulation. The T6S+ division rate *α*_*t*_ is taken as the (inverse) time unit of the system and is set equal to 1. The sensitive division rate *α*_*s*_ is generally set higher than *α*_*t*_, as only T6S+ cells pay the cost of maintaing the T6S. Upon cell division, a copy of the dividing cell is placed in a vacant space adjacent to the dividing cell (Fig 2a). If no vacancies exist adjacent to the dividing cell, nearby cells are pushed out of the way to make room (S1 Text).

Each T6S+ cell has a fixed rate *γ* of initiating an attack (Fig 2b). The attack is then resolved according to an individual-based rule: attack exactly one randomly chosen nearest neighbor if there is one; otherwise do nothing. If the attack targets a sensitive cell or a cell of a different T6S+ strain, the target dies and its lattice site becomes unoccupied; T6S+ cells are immune to attack by cells of the same T6S+ strain, as observed experimentally [26]. The overall rate of events is controlled by the simulation timestep multiplier, *λ* (S1 Text).

## Results

### Competition between T6S+ and sensitive strains

To determine the effect of T6S on multi-species population dynamics, we simulated a competition between T6S+ and sensitive strains during a range expansion. The simulations begin with a well-mixed, fully occupied circular inoculum of approximately 500 individuals (S1 Text). For 2D simulations on a triangular lattice, the starting population is 469 individuals (i.e. inoculum radius *r*_0_ = 12).

The T6S+ division rate is chosen as the unit of time, *α*_*t*_ = 1. The three other parameters are the sensitive strain growth rate *α*_*s*_, the initial sensitive strain fraction, and the attack rate *γ*. (In simulations in which there are no T6S+ cells, the unit of time is *α*_*s*_ = 1.) The attack rate *γ* and the sensitive strain growth rate *α*_*s*_ are found to offset one another as discussed below. The parameter space was extensively explored. Fig 2 shows parameters chosen to emphasize the effect of varying the attack rate *γ* and the initial sensitive strain fraction. Specifically, we fixed the sensitive strain growth rate as *α*_*s*_ = 4 and varied *γ* and the sensitive fraction.

When the attack rate is low (*γ* = 5), sensitive cells can ultimately dominate even when the sensitive strain fraction starts as only a 10% minority (Fig 2c, S2 Video). Initially, the sensitive population declines as isolated individuals are attacked and killed. Eventually, only a small number of surviving sensitive domains remain, concentrated along the periphery of the colony. However, because sensitive cells grow faster than T6S+ cells, these domains begin to outgrow the T6S+ strain, eventually leading to a majority sensitive population. By contrast, at high attack rate (*γ* = 15) and an initial 10% sensitive strain fraction all sensitive individuals are rapidly eliminated (Fig 2d). When the initial sensitive strain fraction is increased to 50%, a larger number of sensitive cells begin near to one another, accelerating the formation of sensitive domains; the early formation of these domains helps the sensitive strain to survive and eventually dominate the T6S+ strain, even under a high rate of attack (Fig 2e and 2f).

An analysis of multiple, independent simulations (Fig 2g) shows that sensitive populations decline and then recover when both the attack rate and initial sensitive strain fraction are low (upper left), or when both are high (lower right). During the period of decline, isolated sensitive cells are eliminated while clusters of sensitive cells enjoy a degree of protection from attack. The monotonic increase of the sensitive population fraction in the most favorable conditions—high initial sensitive strain fraction, low attack rate (lower left)—results from the early formation of sensitive domains, whereas adverse conditions—low initial sensitive strain fraction, high attack rate (upper right)—preclude sensitive domain formation and lead to elimination of the sensitive strain.

### Smallest viable sensitive domain

**Fig 3:**
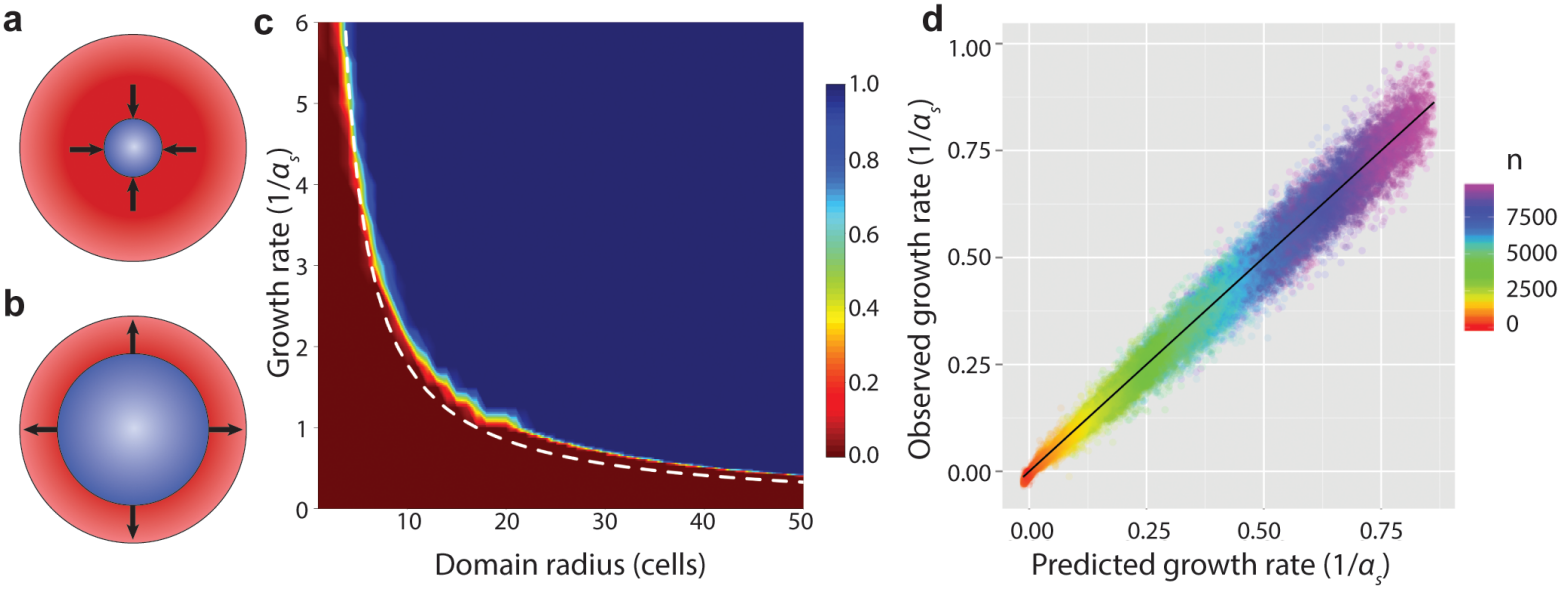
Sensitive T6S- individuals can dominate T6S+ competitors. (a-b) A ball of sensitive cells (blue) is surrounded by a thick layer of T6S+ cells (red). (a) Below a critical radius, the sensitive strain ball tends to shrink to extinction; (b) above it, the ball tends to expand. This behavior is demonstrated for 1D, 2D, and 3D. (c) Heat map of the probability that a 2D sensitive domain surrounded by T6S+ competitors achieves steady growth, as a function of sensitive strain growth rate and initial radius of the sensitive domain. Dashed curve indicates predicted critical parameter values based on Eq. S1. Attack rate $\tilde{\gamma }=8$; interpolated from 80,250 simulations with timestep multiplier *λ* = 500. (Sensitive population either decreased or increased from near the outset of each simulation; consequently, simulations were run only until the sensitive population changed by a factor of three in either direction.) (d) Comparison of growth rate observed in single-domain sensitive 2D growth simulations (*y*-axis) to the values predicted for this regime in Eq 1 (*x*-axis). Points represent the average, by sensitive population, across all simulations with the same parameters (20 per condition; *λ* = 2). Color represents domain radius; black line is *y* = *x*.

Since T6S-mediated killing can take place only at the interface between T6S+ and sensitive strains, we hypothesized that the net growth rate of the sensitive strain depends on the difference between the area or volume of a sensitive domain and the extent of the interface between the strains. To identify the dependence of this relationship on attack rate and relative growth rates, we studied a simple sensitive domain model (Fig 3a and 3b). The 2D simulations begin with a fully-occupied, homogeneous circular sensitive inoculum. As in the competition model, all individuals are capable of cell division. As before, the model assumes that interior cells can push other cells toward the surface of the colony to make room for their daughter cells (S1 Text). To simulate attack, individuals at the outer periphery are subject to being killed at a rate $\tilde{\gamma }$, essentially equivalent to embedding the sensitive domain in a larger T6S+ domain.

To explore the transition from sensitive strain collapse to sensitive strain growth observed in Fig 2, we varied the sensitive strain domain radius while holding constant the “attack” rate $\tilde{\gamma }=8$, retaining the *α*_*s*_ = 4 growth rate from the earlier competitions. Most sensitive strain domains with starting radius *r*_0_ ≤ 5 shrank toward zero, while larger domains survived (S3 Video). We then varied the sensitive strain growth rate, allowing it to fall below *α*_*s*_ = 1. Strikingly, the minimum sensitive strain domain radius required for survival depends inversely on the relative sensitive strain growth rate, implying that a sufficiently large sensitive strain domain can resist displacement by even a faster-growing T6S+ attacker (Fig 3c).

We can readily estimate the critical population size n* above which a sensitive strain domain is expected to enjoy a net positive growth rate. Above this value, a sensitive domain would not shrink as a result of T6S+ competition, although it could, depending on conditions, represent an increasingly small fraction of total population. Eq 1 represents a theoretical “worst-case” scenario for a domain of sensitive cells, in which they are completely surrounded by an infinite domain of T6S+ cells. The key observation is that the rate of killing is proportional to the length of the interface between strains, while the rate of sensitive strain population growth is proportional to the sensitive population. For a population size *n* in 2D, the size of the interface is simply the circumference of the circle. Hence,

$$\frac{dn}{dt}=\alpha_{s}n-2\tilde{\gamma }\;{\left(\pi n\right)}^{\frac{1}{2}}.$$ (1)

Solving Eq 1 for *n* at *dn*/*dt* = 0, i.e. at the unstable fixed point between increasing and decreasing *n*, we find that

$$n^{*}=\frac{4{\tilde{\gamma }}^{2}\pi }{\alpha_{s}^{2}},$$ (2)

which is shown as a dotted line on Fig 3c. The slight divergence at high radius between the predicted and simulated values is the result of accumulated simulation error (S1 Text). The finding suggests that, even at this theoretical limit of maximal contact with T6S+ competitors, a sensitive domain can persist for long times.

Fig 3d shows simulation results for *dn*/*dt* plotted against the prediction from Eq 1. The rate of change of sensitive strain population was measured periodically in simulations with initial domain radii from *r*_0_ = 3 to *r*_0_ = 12. Attack rates ranged from $\tilde{\gamma }=0$ to $\tilde{\gamma }=14$; sensitive strain growth rates ranged from *α*_*s*_ = 1 to *α*_*s*_ = 4. The simulations show excellent agreement with the predicted dynamics (*R* ^2^ > .98), despite deviations of the sensitive domain from a pure circle arising both from the lattice structure and from the stochasticity of the simulations. Similar results are obtained for a corresponding relationship in 1D and 3D (S2 Text).

### Depletion of nutrients

The simulations described so far assume an unlimited supply of nutrients. To determine the effect of nutrient depletion on T6S population growth and competition, we developed a variant of the IBM that incorporates local depletion of nutrients. Even very limited nutrient concentrations still lead to exponential growth during range expansions, resulting in growth and competition dynamics that are nearly identical to those of the unlimited-nutrient case (S3 Text).

### Live-culture competition assay

To validate our simulation results, we inoculated 2.5 *μ*L each of of LacZ- T6S+ *V. cholerae* and LacZ+ T6S- *E. coli* onto X-Gal plates at various dilutions (see “Materials and Methods”). We compared the outcomes of these experiments with simulations for which the growth rates of sensitive and T6S+ cells were matched to those of *E. coli* and *V. cholerae*, respectively. In a preliminary estimate, *E. coli* was observed to grow slightly faster than *V. cholerae* (2.19 *h* ^−1^ vs 2.05 *h* ^−1^), so this difference was also used in the simulations. The simulation attack rate was set to *γ* = 5, which yielded a rough parallel with the experimental images. These simulations were run until the colony had doubled in radius.

**Fig 4:**
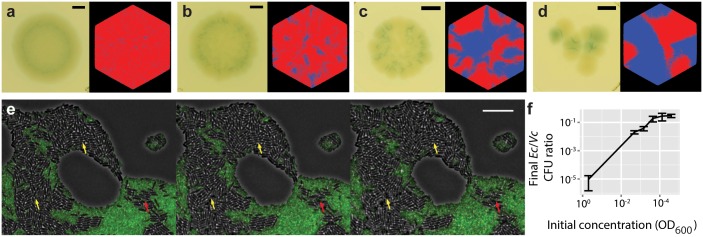
Domain size predicts T6S- survival. (a-d) Comparisons of experimental to simulation outcomes. (a) Left, overnight growth on X-Gal media from an inoculum consisting of *V. cholerae* str. 2740–80 (LacZ-) and *E. coli* MG1655 (LacZ+), starting from equal amounts of OD_600nm_ = 2 × 10^−3^ culture from each species. Right, simulated competition between 6,561 T6S+ individuals and an equal number of sensitive individuals, scattered randomly in an initial domain of *r*_0_ = 82, and allowed to grow until the population radius has doubled (*λ* = 500). (b) 9-fold dilution (experiment and simulation); (c) 81-fold dilution; (d) 729-fold dilution. Scale bars 1mm. (e) Fluorescent micrograph of competition between *E. coli* and *V. cholerae*; shown as illustration of target cell killing. Scale bar 10 *μ*m. Arrows indicate areas of *E. coli* net growth (yellow) and net decline (red). (f) Ratio of *E. coli* to *V. cholerae* CFUs, after overnight growth starting from equal initial amounts, as a function of initial inoculum concentration.

Fig 4a–4d compare the experimental and simulated competitions, with initial inoculum concentrations decreasing 9-fold with each successive panel. As the inoculum becomes more dilute, single-species domains become larger. Simultaneously, *E. coli* become more numerous (Fig 4f; S4 Video). In a micrograph of the experimental competition, large domains of *E. coli* are observed to grow, while smaller domains undergo proportionately higher cell death (Fig 4e). S5 Video suggests that these *E. coli* domains persist stably after 24h. In the simulations, the final sensitive population is seen to increase as initial inoculum density decreases. This is due to the formation of large sensitive domains prior to initial T6S+ encounter, leading to increased sensitive strain survival.

Interestingly, in the low-resolution images, a darkened region is observed along the interspecies interfaces, but not at same-species microcolony interfaces. We infer that the darkened zones represent an accumulation of *E. coli* lysates, due to the continual renewal of the interspecies front by cell division within the bulk.

### T6S+ invasion dynamics

**Fig 5:**
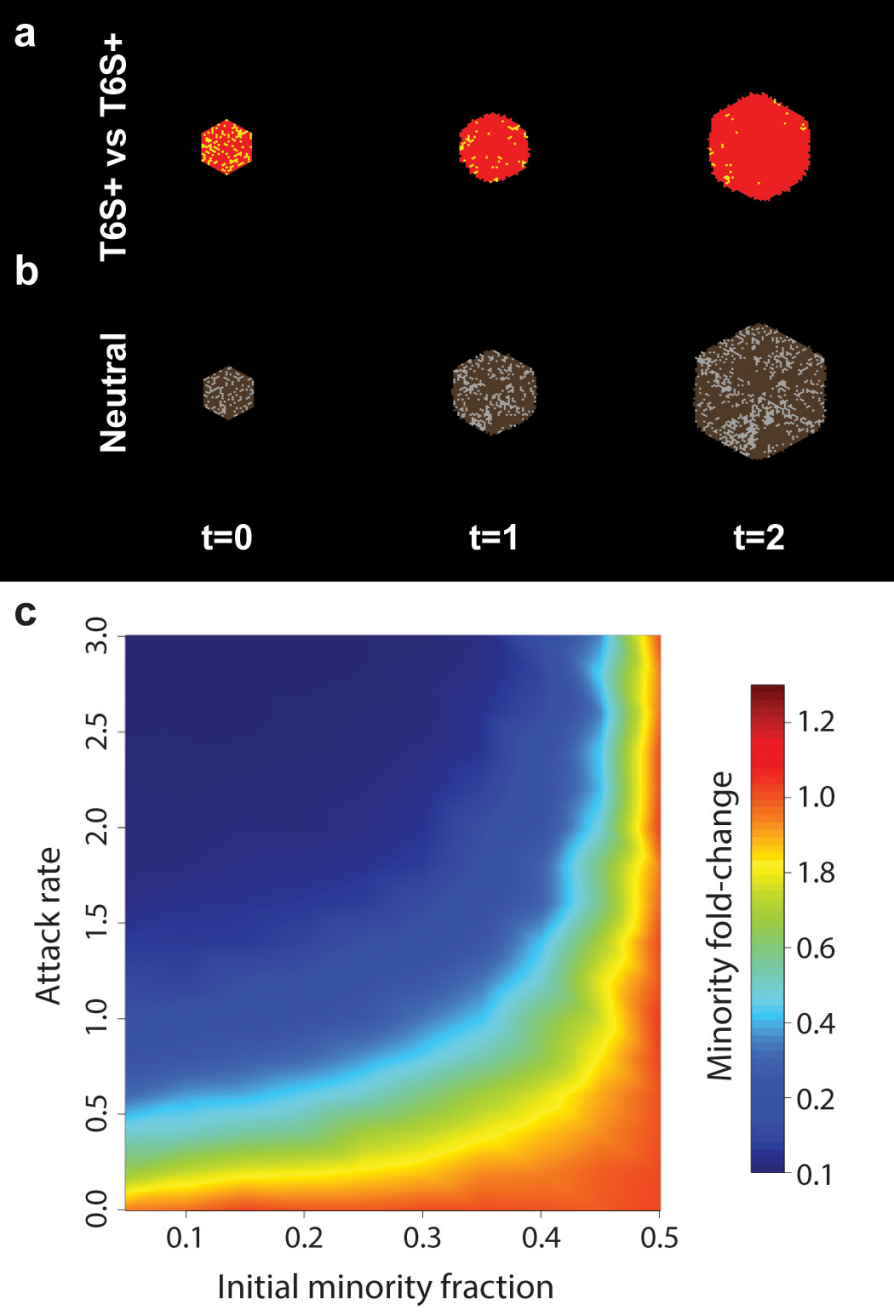
Competition between T6S+ strains. (a-b) Range expansion of two competing T6S+ strains. (a) Each strain kills only individuals of the other type. For each strain, the growth rate is *α*_*t*_ = 1 and the attack rate is *γ* = 2. (b) No killing occurs; grey and brown cells grow neutrally (*γ* = 0) and at the same rate (*α*_*t*_ = 1). Initial inoculum is well-mixed and has radius *r*_0_ = 12; starting minority fraction is 25%. (c) Fold-change in minority fraction, starting at radius *r*_0_ = 12 (*n*_0_ = 469) and growing to exactly 10-fold larger. Orange (fold change = 1.0) indicates that the initial population ratio was retained. Values interpolated from 3,200 simulations. Simulation timestep multiplier *λ* = 1.

So far, we have considered competition between T6S+ and sensitive bacteria. We next investigated whether being T6S+ could help in the case of invasion by a T6S+ competitor. To answer this question, we simulated a competition between two T6S+ strains during a range expansion. Each strain can kill the other, but is immune to self-attack. Each strain has the same attack rate *γ* and cell division rate *α*_*t*_ = 1. Fig 5 shows two T6S+ strains (yellow and red) that were allowed to compete during a range expansion from *n*_0_ = 469 (*r*_0_ = 12) to a final population of *n*_*f*_ = 4690. The relative success of the invasion was measured by comparing the initial yellow (minority) fraction to the final yellow fraction.

In the presence of attack, the minority population is quickly eliminated (Fig 5a). By contrast, in the absence of attack the minority fraction remains roughly constant throughout the course of the range expansion (Fig 5b, S6 Video). As the attack rate increases, the initial minority fraction needed for survival asymptotically approaches 50% (Fig 5c). Note that for equal initial numbers of red and yellow cells, attack leads to spontaneous segregation from a well-mixed inoculum, with higher attack rates leading to faster and more thorough sectoring (S6 Video). Equivalent competitions in 1D and 3D led to analogous results (S4 and S5 Figs). These results imply that T6S+ is useful for defending established populations against invasion.

## Discussion

Gram-negative bacteria can employ T6S to kill competitors, yet the system is not found universally among these bacteria. To better understand the conditions favoring T6S, we modeled a competition between T6S+ and sensitive strains. In a range expansion from a well-mixed inoculum, we found that the sensitive cells can survive in the presence of T6S+ competitors by forming compact domains that protect interior cells from attack. To test these results, we competed T6S+ *V. cholerae* and T6S-, sensitive *E. coli* in an analogous range expansion. We observed that *E. coli* outcompeted *V. cholerae*, so long as the *E. coli* had the opportunity to form compact domains. Finally, we found that in a model competition between two equivalent T6S+ strains the more numerous strain always drove the minority to extinction.

It is informative to compare the current model to related model systems. For example, in a Lotka-Volterra model, a prey species grows in the absence of predation, and a predator grows faster in the presence of prey [48]; such systems have also been generalized to lattices [49]. By contrast, T6S+ does not grow faster as a result of killing, but potentially occupies more of the habitat. In this sense, the current model is more closely analogous to colicin dynamics in *E. coli* [50, 51]. Chao and Levin [52] observed that a colicin-producing strain of *E. coli* dominated a sensitive strain on soft agar by creating a zone of inhibition around itself, preventing the sensitive cells from exploiting the habitat. Colicin dynamics have also been studied using an IBM based on contact-mediated killing [53]. The colicin IBM differs from our T6S model in two respects: in [53], agents can only divide into adjacent vacancies, and sensitive cells have a strict growth advantage. The colicin model predicts that either species can dominate, with dominance depending primarily on parameter choices. By contrast, in the current study, initial colony size determines the survivorship of sensitive cells at all parameter values. The difference comes from the fact that in our model for T6S-mediated competition, interior sensitive cells are protected from killing by the outermost layer of cells. Such a “refuge” effect has previously been studied in the context of predator-prey dynamics, where density-driven sheltering is observed to destabilize predator-prey ratios relative to a well mixed model [54].

Our model employs a number of simplifying assumptions. Most importantly, cells are represented as agents on a regular lattice, and cells divide stochastically. While cell shape can affect the details of colony morphology during range expansions, it does not seem to affect the qualitative population dynamics [55]; indeed, lattice population dynamics have been shown to be consistent with the dynamics of real microbial populations [56]. The similarity of our observations in 1D, 2D, and 3D further suggests that our results are not sensitive to cellular geometry. Similarly only overall growth rates, rather than the detailed timing of cell divisions, are important for long-term population dynamics [55].

It has been hypothesized that nutrient depletion may introduce a substantial advantage for T6S+ strains. In practice, cells at the interior of a natural community face nutrient and oxygen depletion [57]. Does this limitation result in a different competitive outcome? In a simple model of nutrient depletion, we found that a moderately nutrient-limited environment leads to dynamics extremely similar to those in the absence of limitation (S3 Text). This is because exponential growth ensures that only a very small fraction of the population occupies a fully depleted zone (S7 Fig). Thus, our preliminary results suggest that the effects of nutrient depletion on cell growth do not qualitatively alter the population dynamics arising from T6S-mediated competitions. Under special circumstances, such as burrowing invasions of a nutrient-depleted biofilm, T6S-mediated cell lysis could provide a significant nutrient benefit beyond the direct benefit of killing competitor cells. Typically, this effect would be limited, as the nutrient benefit would be divided among both T6S+ species and their prey. In an entirely nutrient-depleted environment, though, actively growing invaders would have an early growth advantage over previously quiescent resident cells.

In determining the ecological role of T6S, the costs of maintaining a T6SS must be taken into consideration. The T6SS requires the expression of 13 core genes, the assembly and disassembly of the baseplate structure and sheath, and the production of the secreted effectors [19, 27, 30]. Immunity to T6S requires the maintenance of a complementary immunity protein, and may require additional modifications to the attacker’s peptidoglycan [26]. Selective use of T6S can mitigate these costs by reducing the frequency of wasteful attacks. To this end, bacteria have evolved a variety of T6SS regulatory schemes, including quorum-sensing and retaliation.

Quorum sensing can reduce wasteful attacks by repressing T6S until it is likely to provide a benefit [21, 58]. For example, QS regulates expression of T6SS in *V. cholerae* [59]. Interestingly, expression of T6SS and natural competence is induced by high cell density and growth on chitinous surfaces, which suggests a role of T6SS in horizontal gene transfer [60]. In addition, the *V. cholerae* QS signal integrates both species-specific and multigeneric signals [61], which means that the presence of competitors could also activate *V. cholerae*’s T6SS. However, reflecting the diversity of T6S roles, T6S is not always upregulated in response to high density. In *P. aeruginosa*, there are three T6SSs; species-specific QS signals LasR and MvfR activate two of these T6SSs, but repress the third [62].

Like quorum sensing, “retaliatory” T6S attack can prevent attack until a hostile cell is encountered. For example, *P. aeruginosa* is observed to engage in retaliatory T6S attack [27, 63, 64]. This ‘tit-for-tat’ strategy could limit wasteful T6S+ interactions within clonal populations, as well as facilitating coexistence within productive consortia. Notably, *P. aeruginosa* also attacks its target repeatedly; by eliminating wasteful attacks, retaliators are also free to employ a more concerted (and damaging) series of attacks [27].

In considering the ecological role of T6S, it is instructive to consider an analogous system found in marine invertebrates. Members of the phylum *Cnidaria*, which includes corals, hydrae, and jellyfish, possess an explosive cell called a nematoycte containing a harpoon-like projectile [65]. Upon detonation, the effector is propelled with extreme force (up to 40,000g) into a target, leading to paralysis and death [66]. Among corals, nematocytes are used interspecifically to compete for habitat access. High attack rates are most commonly observed among slower-growing species, where nematocytes are used to defend against encroachment [67]. Our results suggest that, like nematocytes, T6S can also offset a growth rate disadvantage. The full breadth of its ecological role, however, is only beginning to come into focus.

## Supporting Information

S1 Text Simulation details. (PDF)

S2 Text Generalization of sensitive domain survival to 1D and 3D. (PDF)

S3 Text The impact of nutrient depletion on T6S-mediated population dynamics. (PDF)

S1 Video T6S-mediated interactions between bacteria. Competition between *V. cholerae* str. 2740–80 (sheath labeled with GFP) and *E. coli* MG1655 (unlabeled). 60 frames; frames are 30s apart.
(MP4)

S2 Video A simple spatial model of T6S-driven community dynamics. Time series of simulated competitions between a T6S+ strain (red) and a sensitive strain (blue) during a range expansion in 2D. In all cases the T6S+ growth rate is *α*_*t*_ = 1, and the sensitive strain growth rate is *α*_*s*_ = 4. Initial sensitive strain fractions are 0.1 (upper) and 0.5 (lower). Attack rates are *γ* = 5 (left) and 15 (right). Timestep multiplier *λ* = 1.
(MP4)

S3 VideoCritical domain size for sensitive strain survival.Time series of a simulated range expansion of a sensitive strain subject to stochastic killing at the outer boundary of the colony. Initial colony radius varies from *r*_0_ = 4 (left) to *r*_0_ = 7 (right). The growth rate is *α*_*s*_ = 4 and the killing rate at the outer boundary is $\tilde{\gamma }=8$. Timestep multiplier *λ* = 1.(MP4)

S4 Video Community dynamics between T6S+ and T6S-sensitive populations. Fluorescent micrograph of competition between T6S+ *V. cholerae* and T6S-sensitive *E. coli* (see Fig 4e). two fields, 60 frames; frames are 20s apart. Shown as illustration of target cell killing.
(MP4)

S5 Video Time series of T6S-mediated competition during range expansion. Overnight growth on X-Gal media from an inoculum consisting of *V. cholerae* str. 2740–80 (LacZ-) and *E. coli* MG1655 (LacZ+), starting from equal concentrations of OD_600_ = 0.5 culture from each species. Dilution shown at bottom of each panel. 1 frame = 10 minutes; scale bar = 1mm.
(MP4)

S6 Video Competition between T6S+ strains. Time series of simulated range expansion of two competing T6S+ strains. Initial inoculum is well-mixed and has radius *r*_0_ = 12. Starting minority (yellow) inoculum fraction is 10% (bottom), 25% (middle), and 50% (top). Attack rates are *γ* = 0 (left), *γ* = 1 (middle), and *γ* = 2 (right). Timestep multiplier *λ* = 1.
(MP4)

S1 Fig The effect of time step on simulation error at large population in 1D. (a) Plot of simulated growth rates (y-axis) vs. predicted growth rates from Eq. S1 (x-axis) for a sensitive domain with simulation timestep multiplier *λ* = 0.25. Each point represents the average, over identical conditions, from 5 simulations. (b) The same plot, averaging over 20 simulations with *λ* = 2.
(TIF)

S2 FigSensitive domain growth dynamics in 1D.(a) Comparison of simulation results (y-axis) to predicted values from Eq. S1 (x-axis) for rate of growth of a 1D sensitive domain. Points represent the average, by sensitive population, across all simulations with the same parameters (40 per condition). Color represents domain radius; black line is *y* = *x*. Simulation timestep multiplier *λ* = 0.01. (b) Heat map of the probability that a 1D sensitive domain surrounded by T6S+ competitors achieves steady growth, as a function of sensitive strain growth rate and initial radius of the sensitive domain. Dashed line indicates predicted critical parameter values based on Eq. S1. Attack rate $\tilde{\gamma }=20$; timestep multiplier *λ* = 0.5. Interpolated from 1.9 million simulations.(TIF)

S3 FigSensitive domain growth dynamics in 3D.(a) Comparison of simulation results (y-axis) to predicted values from Eq. S3 (x-axis) for rate of growth of a 3D sensitive domain. Points represent the average, by sensitive population, across all simulations with the same parameters (5 per condition; *λ* = 2.0). Color represents domain radius; black line is *y* = *x*. (b) Heat map of the probability that a 3D sensitive domain surrounded by T6S+ competitors achieves steady growth, as a function of sensitive strain growth rate and initial radius of the sensitive domain. Dashed curve indicates predicted critical parameter values based on Eq. S3. Attack rate $\tilde{\gamma }=8$; interpolated from 6,090 simulations (*λ* = 2000).(TIF)

S4 Fig Range expansion of two competing T6S+ strains. Each strain kills only individuals of the other type; the two strains are otherwise identical. Initial inoculum is well-mixed; starting minority (yellow) fraction is 25%. For each strain, the growth rate is *α*_*t*_ = 1 and the attack rate is *γ* = 2. (a) Kymograph of a 1D competition; time is shown on the x-axis. Initial innoculum *r*_0_ = 500; timestep multiplier *λ* = 1. (b) Center slice through a 3D competition. Initial innoculum *r*_0_ = 6; timestep multiplier *λ* = 2.
(TIF)

S5 Fig Fold-change in minority fraction after 10-fold growth in population of two competing T6S+ strains. For each strain, the growth rate is *α*_*t*_ = 1. (a) Competition in 1D. Initial innoculum *r*_0_ = 500; timestep multiplier *λ* = 1. (b) Competition in 3D. Initial innoculum *r*_0_ = 6; timestep multiplier *λ* = 1.
(TIF)

S6 Fig Time series of nutrient-limited population expansion (*K* = 2). Time points shown are *t* = 0 (left), *t* = 9 (middle), and *t* = 12 (right). Lighter color corresponds to higher nutrient concentration. Simulation scaling factor *λ* = 100.
(TIF)

S7 Fig Nutrient-limited population growth. (a) Population over time for nutrient-limited growth (*K* = 2, blue) and non-limited growth (green). Simulation results shown as solid lines (*n* = 50 per condition, ribbon = 1 S.E.); numerical estimate for deterministic exponential growth (Eq. S11 for limited case, simple exponential growth for non-limited) shown as dashed lines. (b) Long-time inactive fraction as a function of division capacity *K*. Black points: final inactive fraction after range expansion from single cell to radius *r* = 164 (*n* = 10 per condition, bar = 1 S.E.). Green line: numerical estimate (from Eq. S11 and S21) for deterministic growth. Red line: analytical prediction (Eq. S25). For all simulations, scaling factor *λ* = 100.
(TIF)

S8 Fig Nutrient limitation does not qualitatively alter dynamics of simulated T6S-mediated competition. Populations begin with an equal number of T6S+ and sensitive individuals at a specified per-species population, scattered over an *r*_0_ = 84 domain, and grow until the radius has doubled. (a) Population over time for nutrient-limited growth (*K* = 2, left) and non-limited growth (right). Error ribbons smaller than data curve. (b) Mean sensitive fraction over time for nutrient-limited growth (*K* = 2, left) and non-limited growth (right). For both panels, *n* = 40 per condition; scaling factor *λ* = 100. Ribbons = 1 S.E.
(TIF)

S9 Fig Effect of initial T6-sensitive cluster size on dynamics of simulated T6S-mediated competition. Initial populations are placed in compact groups of *m* = 1, 3, or 7 individuals, and with strict separation between these clusters. Shown is final sensitive fraction as a function of initial per-species count. Populations begin with a specified per-species population, scattered over an *r*_0_ = 84 domain, and grow until the radius has doubled. *n* = 90 per condition; scaling factor *λ* = 100. Error bars = 1 S.E.
(TIF)

S1 Table Simulation geometries for range expansions. For range expansion simulations, all cells within a specified Manhattan distance (“Innoculum radius”) are included in the founding population. The resulting population (“Innoculum population”) depends on the lattice geometry.
(PDF)

S2 Table Simulation behavior definitions. (PDF)

S3 Table Parameter ranges for comparison of predicted to simulated rates of sensitive strain growth. (PDF)

S4 Table Active population growth rates for various division capacities. (PDF)
