# Supplementary material for: Established Microbial Colonies Can Survive Type VI Secretion Assault
Source: PLoS Comput Biol. 2015 Oct 20;11(10):e1004520. doi: 10.1371/journal.pcbi.1004520 (PMC4619000; doi:10.1371/journal.pcbi.1004520)
Supplement: S1 Text — (PDF) [file pcbi.1004520.s001.pdf]

Simulation details

Lattice geometries

Simulations were run in a 1D linear, 2D triangular, or 3D cubic lattice. Founding populations were well-mixed and occupied all positions within a specified Manhattan radius of the center point. Unless otherwise specified, the founding population size was chosen to be close to $n_0 = 500$ (Table S1). Each lattice was much larger than required for the maximum final population, such that the population never reached the boundary.

Cell displacement

Our model makes the simplifying assumption that all cells can divide (Fig. 2a). If a cell has one or more adjacent vacancies, it will randomly select one of these to divide into. If all neighbor sites are occupied, then one of the occupied neighbors is chosen at random. Next, the nearest vacancy to the original location is located as is the nearest vacancy to the chosen neighbor, as measured by Manhattan distance. If the original cell is closer to a vacancy, the cells along a randomly-selected shortest path from the original cell to the vacancy are pushed leaving the vacancy occupied; otherwise, cells are pushed in a similar way from the chosen neighbor to the nearest vacancy. If both the original cell and the chosen neighbor are equidistant to a vacancy, the tie is broken at random. The benefit of this approach is that it respects the “inertia” of existing cells, while introducing some nondeterminism in order to reduce artifacts from the regular lattice.

Events

Every cell in the system has a number of “behaviors” or events (Table S2). Behaviors may be probabilistic, meaning that sometimes nothing may happen when the event is triggered. During each simulation step, each cell is instructed to trigger its “attack” behavior. The attack events are resolved one at a time in a random order. The remaining cells are then instructed to trigger their “divide” behaviors. Finally, the state is compared to the halt condition(s) for the simulation (specified population reached, specified cell type constitutes a defined fraction of the population etc.). Images and data points are taken before attack events take place.

Integration error

The base unit of time in the simulations is the generation time for a T6S+ strain, defined as $\tau = 1/\alpha_t$. (In simulations with only sensitive individuals, the unit of time $\tau = 1/\alpha_s$.) The definition of the T6S+ generation time implies that, in each time unit, on average every T6S+ individual will divide once. As with many agent-based simulation schemes, special consideration is needed to resolve simultaneous events in a manner that minimizes error. To this end, we employ a null-event scheme [1] in which, each time the simulation updates its state, time is advanced only a fraction of a step. In each of these simulation updates, the system time advances by only a fraction of τ.

To implement this scheme, we first scale the probability of events from probability per τ to probability per simulation update. We define the interval between simulation updates as $\Delta t = \tau\lambda/N$, where N is the total number of lattice positions and λ is a

scaling factor used to control simulation speed and divergence from an "ideal" simulation with perfect separation of events (i.e., no integration error). If $\lambda = 1$ for example, then a lattice that is entirely full of T6S+ cells would have an average of one cell division per simulation update cycle. The parameter λ represents a tradeoff between the speed at which simulations can be run on a computer and the error due to overlapping events. That is, integration error increases with the value of λ (Fig. S1).

Depending on the system configuration, integration error can create an advantage either for a T6S+ or a sensitive strain. As discussed above, every cell is triggered to consider attacking; once these events are resolved, the cells are triggered to consider dividing. In situations with extensive surface area between strains, therefore, $\lambda \gg 1$ would result in extensive killing prior to cell division, creating an advantage for T6S+ strains. However, when large sensitive domains exist, high λ leads to many individuals dividing before they are at risk of being killed; the result is an advantage to sensitive strains.

Using $\lambda > 1$ allowed us to run large-scale simulations at high replicate. Whenever $\lambda > 1$ was used, we first tested the dynamics at smaller scale with $\lambda = 1$ to verify that the observed results were consistent. In all cases, dynamics were qualitatively equivalent to both smaller scale tests and to analytical predictions.

References

1. Chaterjee A, Vlachos D G (2007) An overview of spatial microscopic and accelerated kinetic Monte Carlo methods. *J Computer-Aided Mater. Des.* 14:253-308
