# Supplementary material for: Established Microbial Colonies Can Survive Type VI Secretion Assault
Source: PLoS Comput Biol. 2015 Oct 20;11(10):e1004520. doi: 10.1371/journal.pcbi.1004520 (PMC4619000; doi:10.1371/journal.pcbi.1004520)
Supplement: S2 Text — (PDF) [file pcbi.1004520.s002.pdf]

Generalization of sensitive domain survival to 1D and 3D

Critical sensitive domain in 1D

Given a linear sensitive domain embedded in an arbitrarily large, 1D T6S+ population, the minimum surviving sensitive domain population n^* is found in a manner analogous to that of a circular sensitive domain in 2D. The birth rate of the sensitive strain scales like the length of the line, and the death rate (due to killing at the interface) is $2\tilde{\gamma}$. Hence the total growth rate is

$$\frac{dn}{dt} = \alpha_s n - 2\tilde{\gamma}. \tag{S1}$$

The observed value of dn/dt is plotted against the predicted value in Fig. S2a; the parameter range is given in Table S3. Solving Eq. S1 for n^*, we find that

$$n^* = \frac{2\tilde{\gamma}}{\alpha_s}, \tag{S2}$$

which is shown as a dashed curve on Fig. S2b.

Critical sensitive domain in 3D

Given a spherical sensitive domain embedded in an arbitrarily large 3D T6S+ population, the minimum surviving sensitive domain population n^* is found in a manner analogous to that of a circular sensitive domain in 2D. We assume that the sensitive domain is spherical. The birth rate of the sensitive strain scales like the total volume of this sphere (i.e., n), and the death rate (due to killing at the interface) scales like its surface area. Hence the total growth rate is

$$\frac{dn}{dt} = \alpha_s n - \tilde{\gamma}\pi^{1/3}(6n)^{2/3}. \tag{S3}$$

The observed value of dn/dt is plotted against the predicted value in Fig. S3a; the parameter range is given in Table S3.

We can predict the transition from sensitive strain extinction to survival. Solving Eq. S3 for n at $dn/dt = 0$, we find that

$$n^* = \frac{36\pi\tilde{\gamma}^3}{\alpha_s^2}, \tag{S4}$$

which is shown as a dashed curve on Fig. S3b.

Mean-field model

The simulations and experiments described in the paper suggest that sensitive domains tend to survive encounters with T6S+ attackers provided those domains have reached a sufficient size. At a population level, this phenomenon is a simple consequence of geometry: population scales with the volume of the sensitive domain, while killing scales only with the surface area. At the scale of individuals, however, the probability of sensitive strain survival is determined by the fraction of neighbors that are capable of

T6S attack. It is therefore useful to consider the conditions required for sensitive strain dominance in the absence of spatial structure. To this end, we consider a mean-field model for a well-mixed system.

Assume that each cell has some fixed number of neighbors z, but that these neighbors are drawn at random from a total population with fraction φ_t of T6S+ individuals and fraction φ_s of sensitive individuals, such that $\varphi_t + \varphi_s = 1$.

The net growth rate for a sensitive individual, i.e. the bare growth rate minus the rate of being attacked and killed depends on the number of T6S+ neighbors. Since each T6S+ individual can attack any of its z neighbors, for a sensitive cell the expected rate of being attacked and killed per T6S+ neighbor is γ/z. The expected growth rates for each type as a function of its randomly sampled neighborhood is

$$g_t = \alpha_t, \tag{S5}$$

$$g_s = \alpha_s - \gamma\varphi_t. \tag{S6}$$

The difference in growth rate between the two strains is

$$\Delta g = \alpha_s - \alpha_t - \gamma\varphi_t. \tag{S7}$$

Sensitive strains are expected to grow more quickly than T6S+ individuals when $\Delta g > 0$, i.e. when

$$\frac{\alpha_s - \alpha_t}{\gamma} > \varphi_t. \tag{S8}$$

If one assumes T6S to incur a non-zero cost, such that $\alpha_s - \alpha_t > 0$, the mean-field model implies that sensitive strains grow faster than T6S+ strains so long as the T6S+ fraction φ_t is not too large. This result holds for a system of any dimensionality. The spatial structure of populations exaggerates this effect by decreasing the probability of sensitive individuals having T6S+ neighbors. Space also allows an arbitrarily small global fraction of the sensitive strain to survive, so long as they have a sufficiently large local population somewhere in the community. For this reason, the spatial model allows sensitive domains to survive attack even if they grow more slowly than T6S+ competitors, whereas this is not possible in the mean-field model.
