# Supplementary material for: Established Microbial Colonies Can Survive Type VI Secretion Assault
Source: PLoS Comput Biol. 2015 Oct 20;11(10):e1004520. doi: 10.1371/journal.pcbi.1004520 (PMC4619000; doi:10.1371/journal.pcbi.1004520)
Supplement: S3 Text — (PDF) [file pcbi.1004520.s003.pdf]

The impact of nutrient depletion on T6S-mediated population dynamics

Nutrient depletion model

Microbial communities deplete nutrients from their environment as they grow. To explore the impact of nutrient depletion, we studied a simple resource-limited variant of our competition model. The variant is identical to the original model except that we now assume each lattice site starts with a finite nutrient supply, and thus a finite "division capacity" K. Every time a cell divides at a particular location, the nutrient supply k at that site is decreased by one unit: $k \to k-1$. Once the local nutrient supply reaches zero, no further cell divisions can take place at that location (Fig. S6). Thus every lattice site can support exactly K cell-division events.

Analysis of growth dynamics

To understand the effect of resource depletion on T6S-mediated competition, it is first helpful to understand its effect on growth in the absence of killing. In this case, each cell division leads to exactly one cell being placed in a previously unoccupied lattice site. By assumption, this newly occupied lattice site has K cell divisions remaining. All other previously occupied lattice sites remain occupied; individual cells may change lattice sites, but overall there is the same number of sites with k cell divisions remaining, except for the new site with K divisions remaining, and the site of the cell-division event, for which $k \to k-1$.

We ignore the case of $K = 1$ (i.e., fixed active population), which implies linear population growth. For all $K > 1$, we can classify all occupied lattice sites by the number of cell divisions remaining. We can then stratify the cell population by the remaining capacity of the site each cell occupies. Let p_k represent the number of cells that occupy lattice sites with k cell divisions remaining, and let us consider the average behavior of p_k.

Let α be the growth rate for cells, i.e. cells on sites with $k > 0$ divide at a rate α. The population of cells on new, capacity K sites increases due to all cell divisions, but decreases due to division of cells on capacity K sites. Hence, on average,

$$\frac{dp_K}{dt} = \alpha \sum_{k=1}^{K-1} p_k. \tag{S9}$$

The populations of all other cell classes p_k likewise decrease due to their own divisions and increase due to divisions of the next higher class p_{k+1}:

$$\frac{dp_k}{dt} = \alpha \left(p_{k+1} - p_k\right), \qquad 0 < k < K. \tag{S10}$$

Setting aside depleted sites (p_0) for the moment, this relation can be expressed by the matrix equation

$$\frac{d\mathbf{p}}{dt} = L\mathbf{p}, \tag{S11}$$

where $\mathbf{p} = (p_1, p_2, \ldots, p_K)$ and

$$L = \begin{pmatrix} -\alpha & \alpha & \ldots & 0 & 0 & 0 \\ \vdots & \vdots & \ddots & \vdots & \vdots & \vdots \\ 0 & 0 & \ldots & -\alpha & \alpha & 0 \\ \alpha & \alpha & \ldots & \alpha & \alpha & 0 \end{pmatrix}. \tag{S12}$$

We observe numerically that the matrix L always has exactly one eigenvalue with positive real part (Table S4). The growth of the population is therefore driven by this dominant eigenvalue. To understand the dynamics of the system, therefore, we seek a positive eigenvalue λ of L such that

$$\frac{d\mathbf{p}}{dt} = \lambda\mathbf{p}. \tag{S13}$$

We further simplify the system by factoring out the growth rate α, defining $\hat{L} = L/\alpha$ and $\mu = \lambda/\alpha$, such that

$$\hat{L}\mathbf{p} = \mu\mathbf{p}. \tag{S14}$$

We make the ansatz that at long times the population structure approaches a geometric series

$$\mathbf{p} = \left(1,\ \beta,\ \ \ldots, \beta^{k-2}, \beta^{k-1}\right). \tag{S15}$$

Substituting this ansatz into Eq. S14, we obtain the relation

$$\begin{pmatrix} -1 & 1 & \ldots & 0 & 0 & 0 \\ \vdots & \vdots & \ddots & \vdots & \vdots & \vdots \\ 0 & 0 & \ldots & -1 & 1 & 0 \\ 1 & 1 & \ldots & 1 & 1 & 0 \end{pmatrix} \begin{pmatrix} 1 \\ \beta \\ \vdots \\ \beta^{k-2} \\ \beta^{k-1} \end{pmatrix} = \mu \begin{pmatrix} 1 \\ \beta \\ \vdots \\ \beta^{k-2} \\ \beta^{k-1} \end{pmatrix}. \tag{S16}$$

For the last line (corresponding to p_K), we have

$$1 + \sum_{k=1}^{K-2} \beta^k = \mu\beta^{K-1}, \tag{S17}$$

and for all other lines we obtain

$$\beta = \mu + 1. \tag{S18}$$

Substituting Eq. S18 into Eq. S17, we obtain the polynomial expression for the eigenvalues of $\hat{L}$

$$1+\sum_{k=1}^{K-2}\left(\mu+1\right)^{k}=\mu\left(\mu+1\right)^{K-1}. \tag{S19}$$

The largest root of Eq. S19 (by real value) is shown in Table S4. The table also shows the dominant eigenvalues of the matrix $\hat{L}$, demonstrating agreement between the two, and confirming the ansatz (Eq. S15) for the asymptotic population structure.

We observe from Table S4 that μ approaches 1 with with increasing K. We therefore write $\mu = 1-\epsilon$ and expand Eq. S19 to obtain the estimate

$$\mu(K)\approx 1-2^{-K}. \tag{S20}$$

Turning our attention to the inactive population p_0, we note that p_0 grows as a result of cell division by subpopulation p_1. Since p_1 divides at a rate α, we have

$$\frac{dp_0}{dt}=\alpha p_1. \tag{S21}$$

At long times, p_1 grows at a rate set by the dominant eigenvalue. At long times, therefore,

$$\frac{dp_1}{dt}\longrightarrow \lambda p_1. \tag{S22}$$

By inspection of these two equations, we observe that for long times

$$\frac{dp_0}{dt}\longrightarrow \frac{\alpha}{\lambda}\frac{dp_1}{dt}, \tag{S23}$$

which implies

$$p_0\longrightarrow \frac{\alpha}{\lambda}p_1=\frac{1}{\mu}p_1. \tag{S24}$$

Substituting Eq. S20 and the ansatz from Eq. S15 into Eq. S24 and simplifying, we obtain the estimate

$$p_0=\frac{1}{2^K}. \tag{S25}$$

Fig. S7a shows predicted and observed population growth over time, both without and with nutrient depletion ($K = 2$). As predicted, nutrient-limited populations grow exponentially, albeit at a slower rate than non-limited populations. The inactive population fraction asymptotically approaches zero as K increases, as predicted by Eq. S25 (Fig. S7b).

Effects of nutrient depletion on T6S competition dynamics

Fig. S8 presents the results of competition between T6S+ and sensitive individuals during a range expansion. The initial conditions and parameters are identical to those used for Figs. 4a-d. In the absence of nutrient limitation, the overall population grows more quickly than when nutrients are limited (Fig. S8a). Nevertheless, even in a

strongly nutrient-limited case ($K = 2$), the qualitative dynamics of the competition are essentially identical whether nutrients are limited or not (Fig. S8b).

Since nutrients are depleted from the inside of a colony outward, might varying the initial microcolony size reveal differences between nutrient-limited and non-limited conditions? Fig. S9 explores competitive dynamics in range expansions for which the cells are initially dispersed into clusters of a specific size. Larger clusters provide an advantage to sensitive cells by giving them more time to form large domains before T6S assault. However, no qualitative difference was observed between nutrient-limited and non-limited conditions. Intuitively, because of exponential growth, only a small fraction of the population is ever in the p_0 state for which division is not possible, so the effects of nutrient depletion are small.
