# Supplementary figures and images for: Established Microbial Colonies Can Survive Type VI Secretion Assault
Source: PLoS Comput Biol. 2015 Oct 20;11(10):e1004520. doi: 10.1371/journal.pcbi.1004520 (PMC4619000; doi:10.1371/journal.pcbi.1004520)

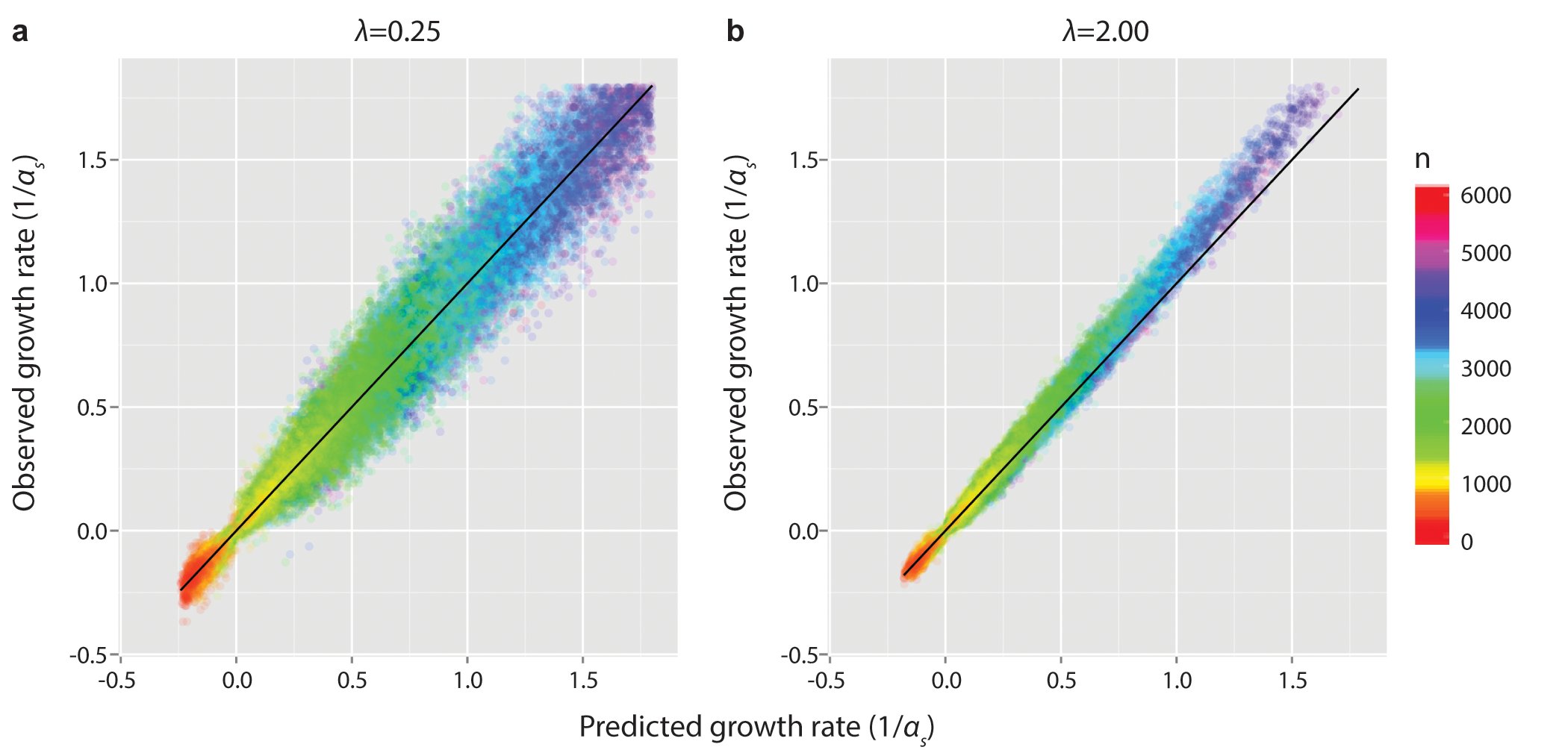

Supplement: S1 Fig — (a) Plot of simulated growth rates (y-axis) vs. predicted growth rates from Eq. S1 (x-axis) for a sensitive domain with simulation timestep multiplier λ = 0.25. Each point represents the average, over identical conditions, from 5 simulations. (b) The same plot, averaging over 20 simulations with λ = 2. (TIF) [file pcbi.1004520.s010.tif]

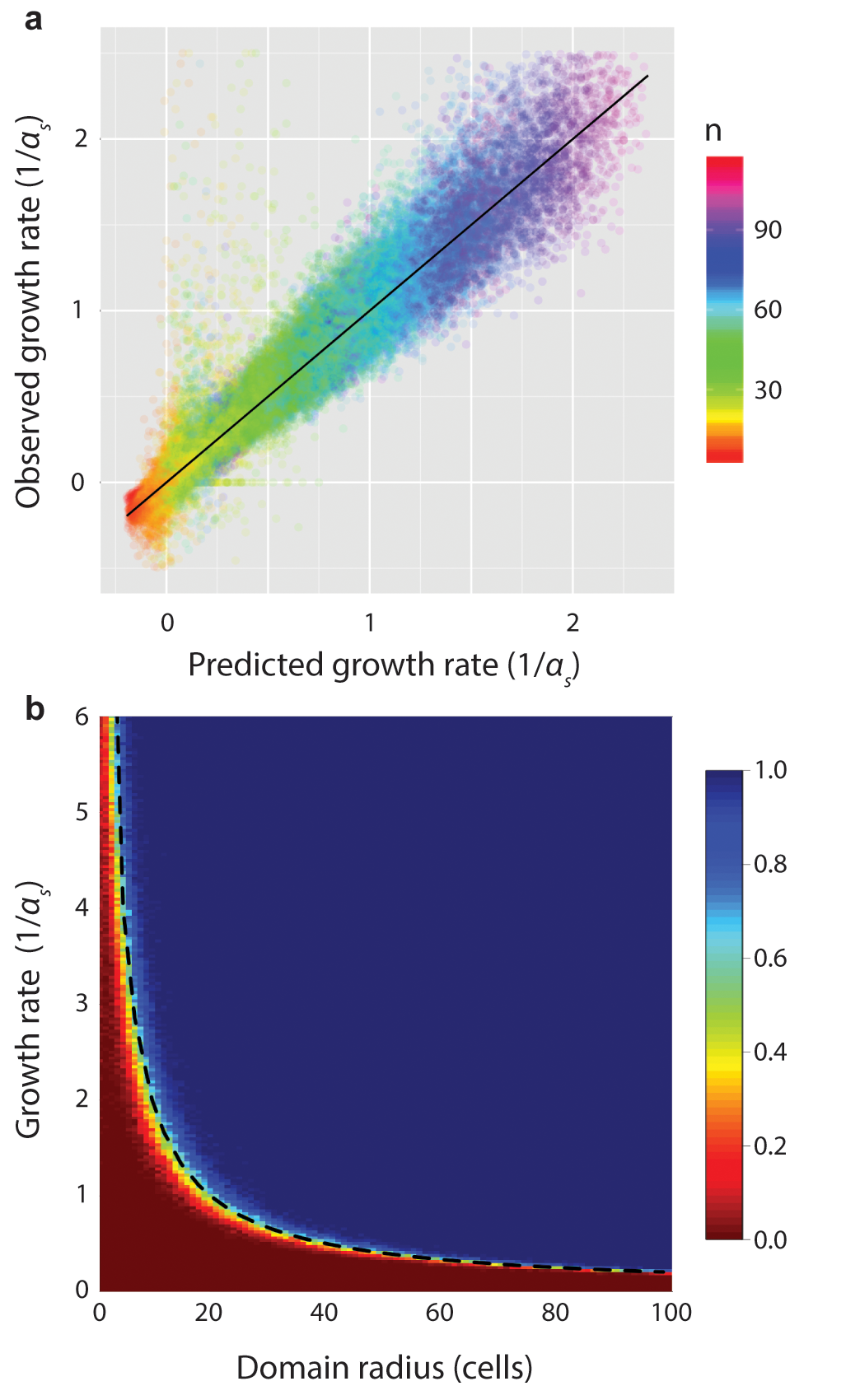

Supplement: S2 Fig — (a) Comparison of simulation results (y-axis) to predicted values from Eq. S1 (x-axis) for rate of growth of a 1D sensitive domain. Points represent the average, by sensitive population, across all simulations with the same parameters (40 per condition). Color represents domain radius; black line is y = x. Simulation timestep multiplier λ = 0.01. (b) Heat map of the probability that a 1D sensitive domain surrounded by T6S+ competitors achieves steady growth, as a function of sensitive strain growth rate and initial radius of the sensitive domain. Dashed line indicates predicted critical parameter values based on Eq. S1. Attack rate γ˜=20; timestep multiplier λ = 0.5. Interpolated from 1.9 million simulations. (TIF) [file pcbi.1004520.s011.tif]

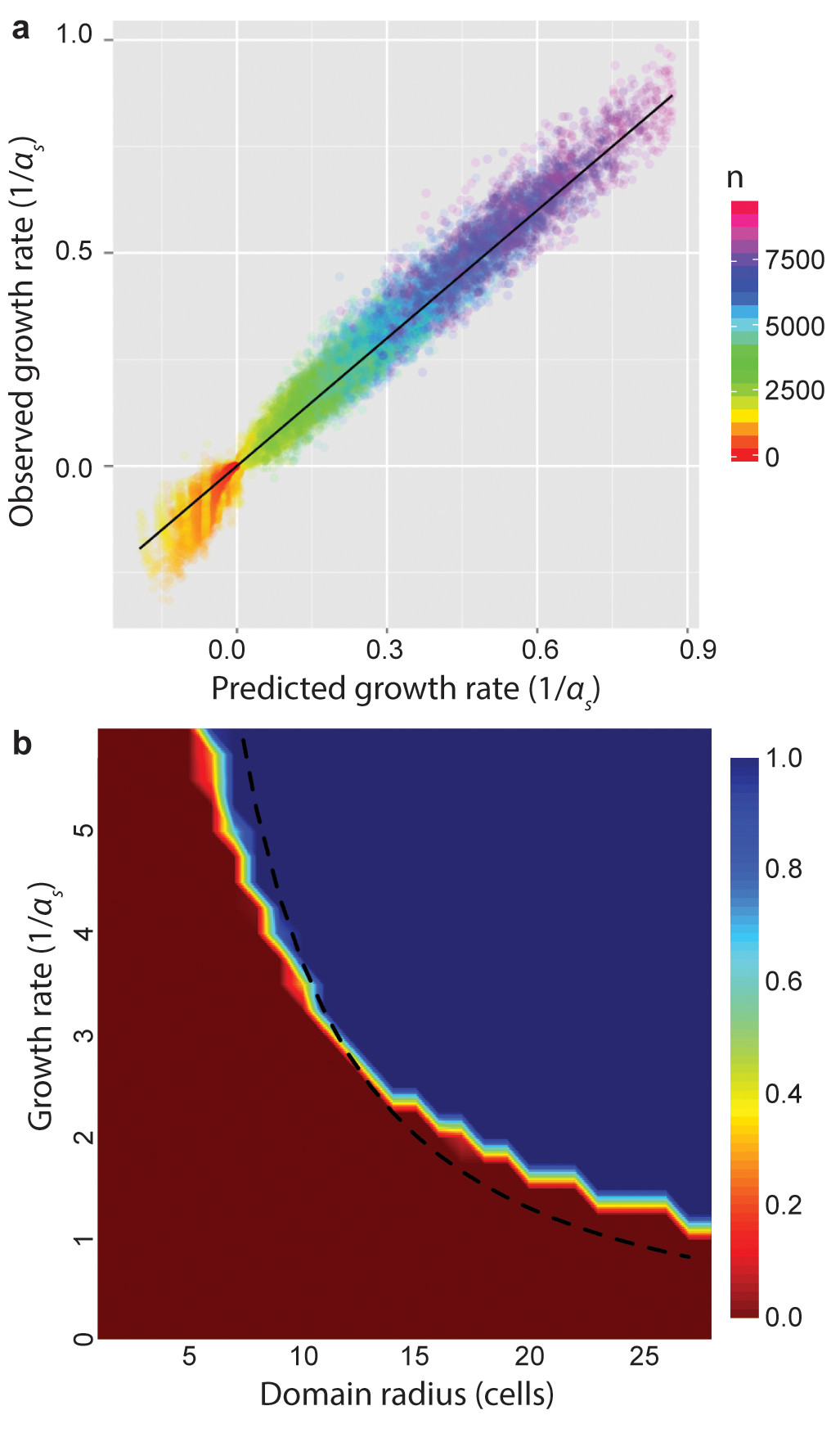

Supplement: S3 Fig — (a) Comparison of simulation results (y-axis) to predicted values from Eq. S3 (x-axis) for rate of growth of a 3D sensitive domain. Points represent the average, by sensitive population, across all simulations with the same parameters (5 per condition; λ = 2.0). Color represents domain radius; black line is y = x. (b) Heat map of the probability that a 3D sensitive domain surrounded by T6S+ competitors achieves steady growth, as a function of sensitive strain growth rate and initial radius of the sensitive domain. Dashed curve indicates predicted critical parameter values based on Eq. S3. Attack rate γ˜=8; interpolated from 6,090 simulations (λ = 2000). (TIF) [file pcbi.1004520.s012.tif]

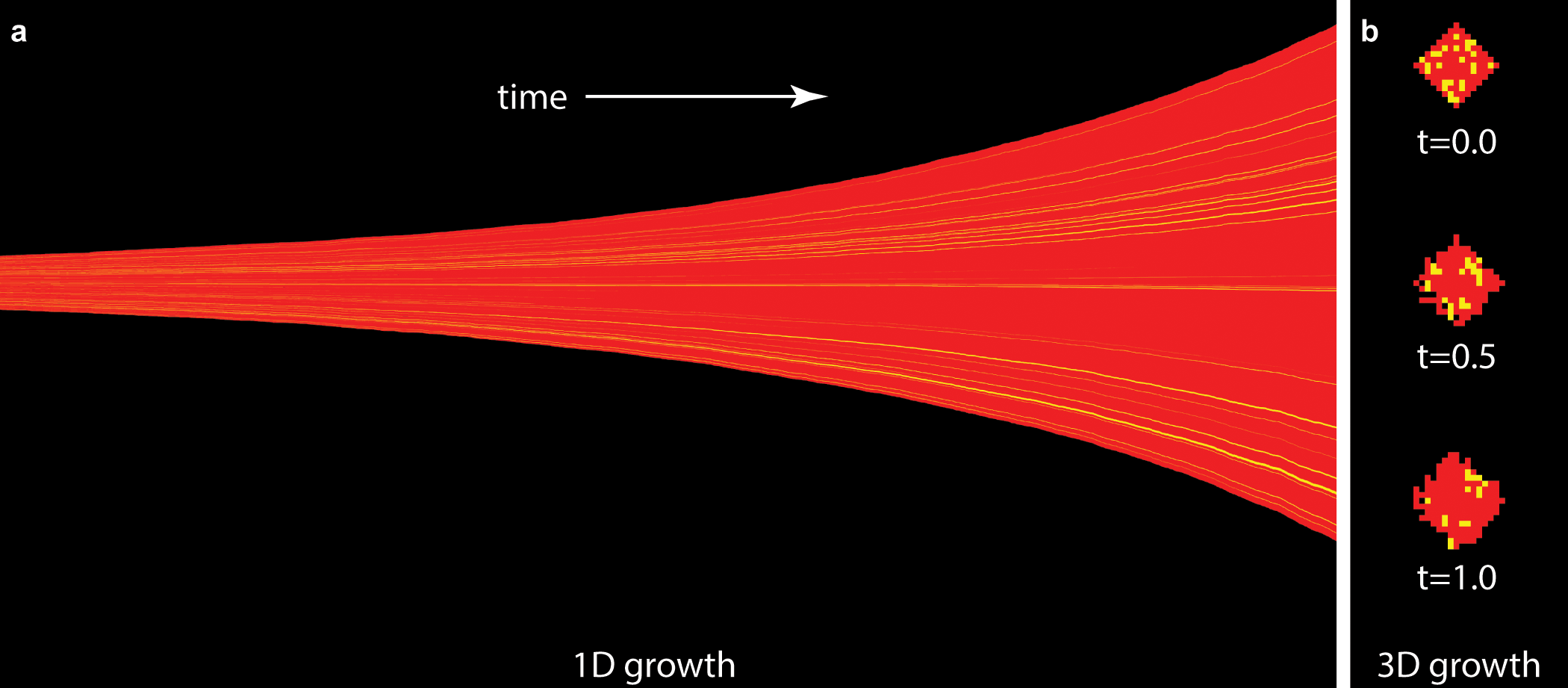

Supplement: S4 Fig — Each strain kills only individuals of the other type; the two strains are otherwise identical. Initial inoculum is well-mixed; starting minority (yellow) fraction is 25%. For each strain, the growth rate is α t = 1 and the attack rate is γ = 2. (a) Kymograph of a 1D competition; time is shown on the x-axis. Initial innoculum r 0 = 500; timestep multiplier λ = 1. (b) Center slice through a 3D competition. Initial innoculum r 0 = 6; timestep multiplier λ = 2. (TIF) [file pcbi.1004520.s013.tif]

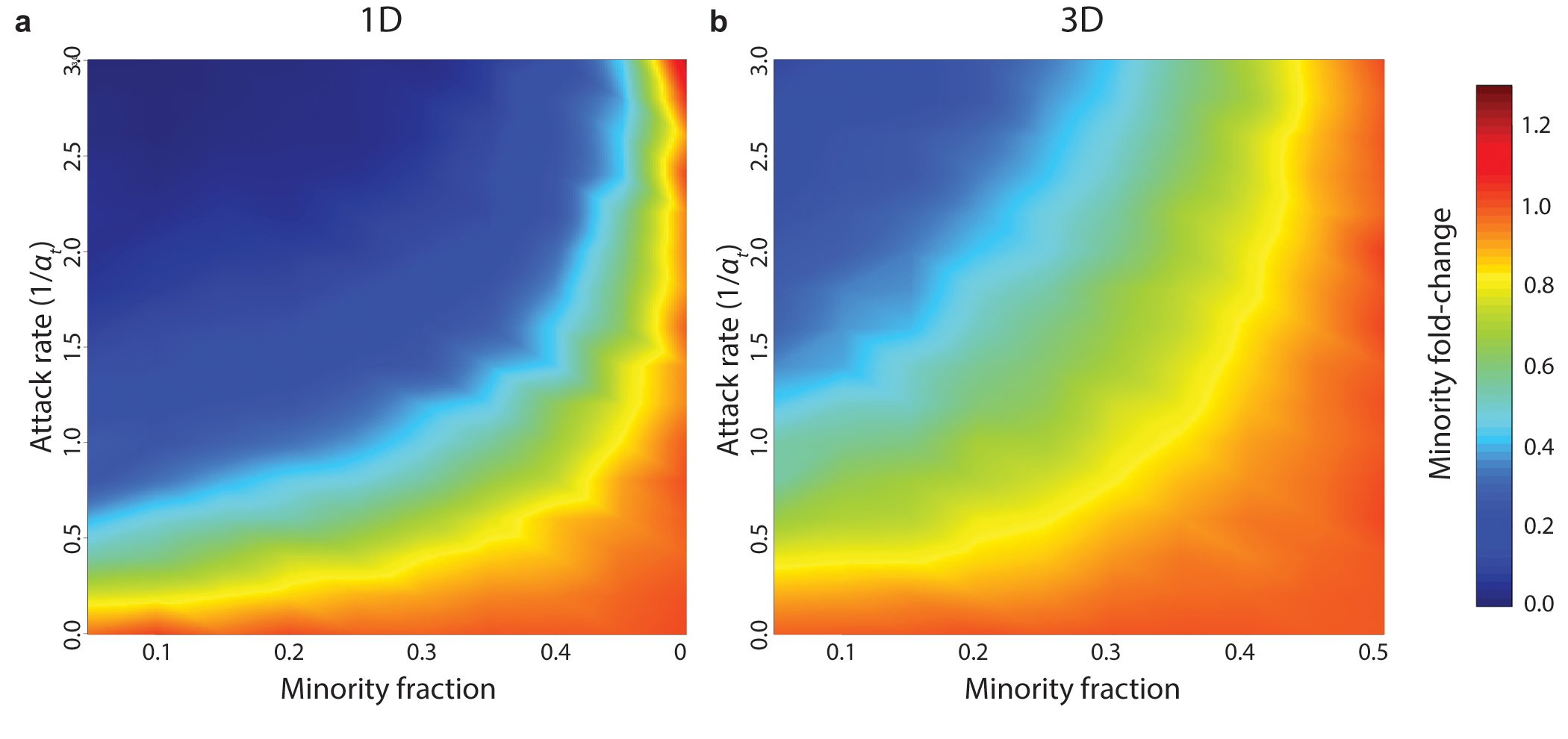

Supplement: S5 Fig — For each strain, the growth rate is α t = 1. (a) Competition in 1D. Initial innoculum r 0 = 500; timestep multiplier λ = 1. (b) Competition in 3D. Initial innoculum r 0 = 6; timestep multiplier λ = 1. (TIF) [file pcbi.1004520.s014.tif]

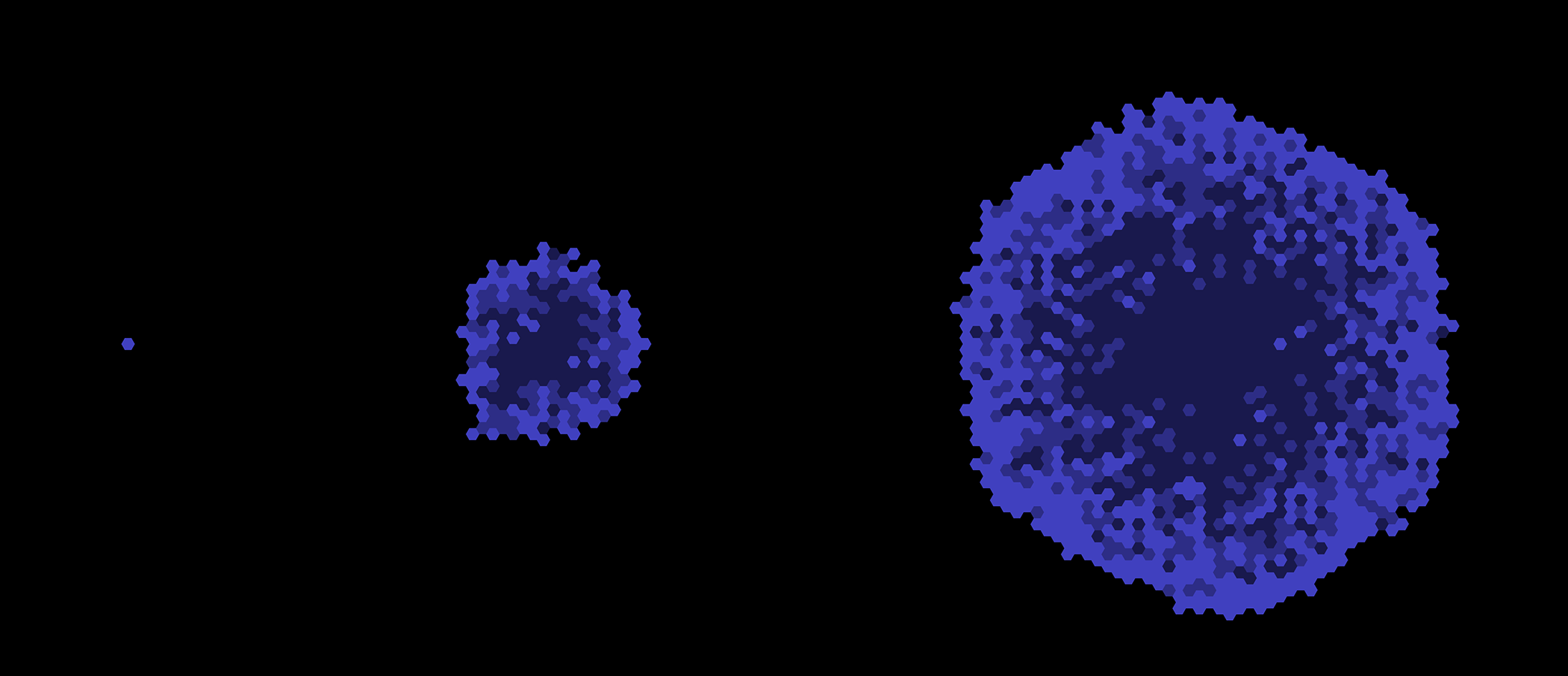

Supplement: S6 Fig — Time points shown are t = 0 (left), t = 9 (middle), and t = 12 (right). Lighter color corresponds to higher nutrient concentration. Simulation scaling factor λ = 100. (TIF) [file pcbi.1004520.s015.tif]

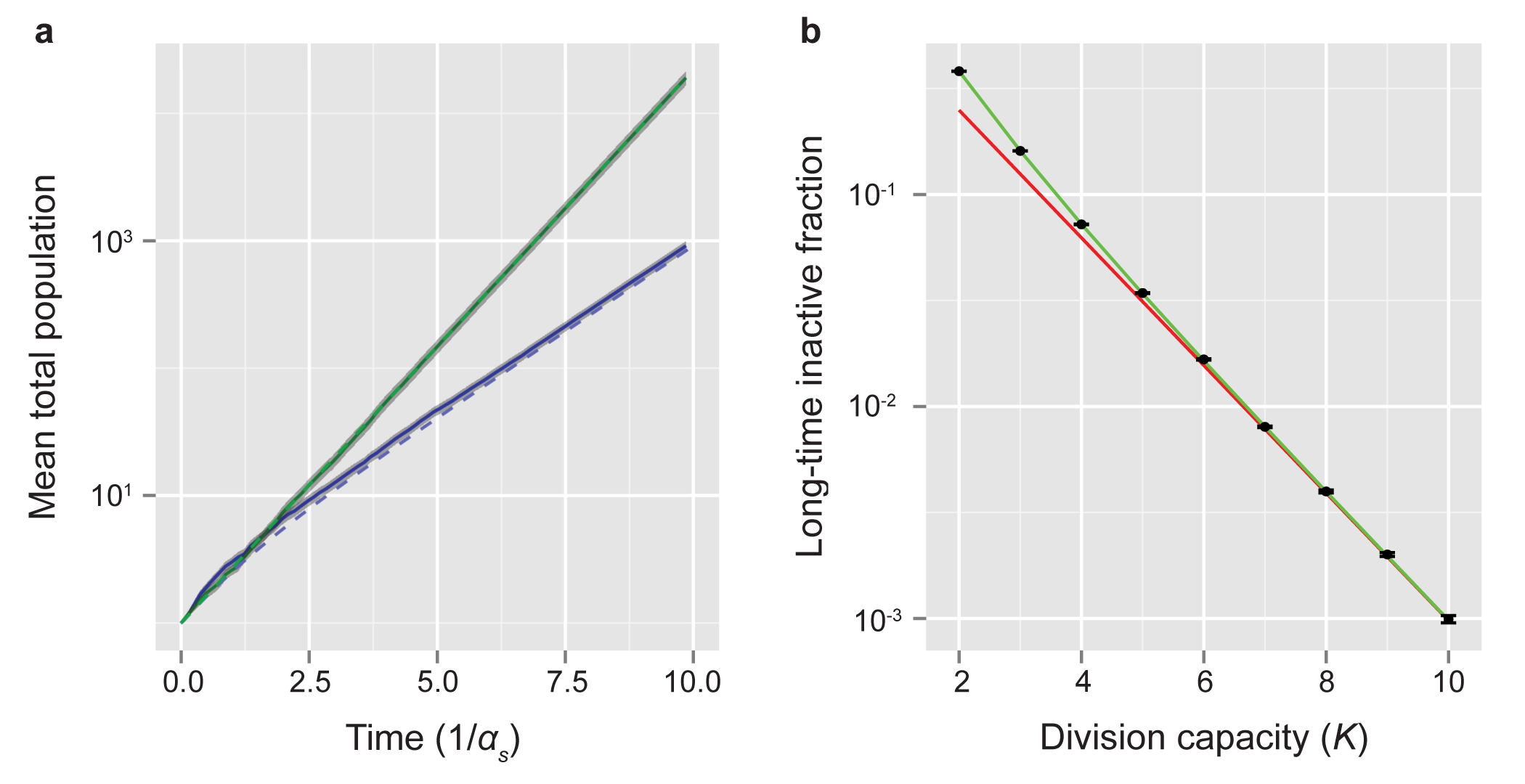

Supplement: S7 Fig — (a) Population over time for nutrient-limited growth (K = 2, blue) and non-limited growth (green). Simulation results shown as solid lines (n = 50 per condition, ribbon = 1 S.E.); numerical estimate for deterministic exponential growth (Eq. S11 for limited case, simple exponential growth for non-limited) shown as dashed lines. (b) Long-time inactive fraction as a function of division capacity K. Black points: final inactive fraction after range expansion from single cell to radius r = 164 (n = 10 per condition, bar = 1 S.E.). Green line: numerical estimate (from Eq. S11 and S21) for deterministic growth. Red line: analytical prediction (Eq. S25). For all simulations, scaling factor λ = 100. (TIF) [file pcbi.1004520.s016.tif]

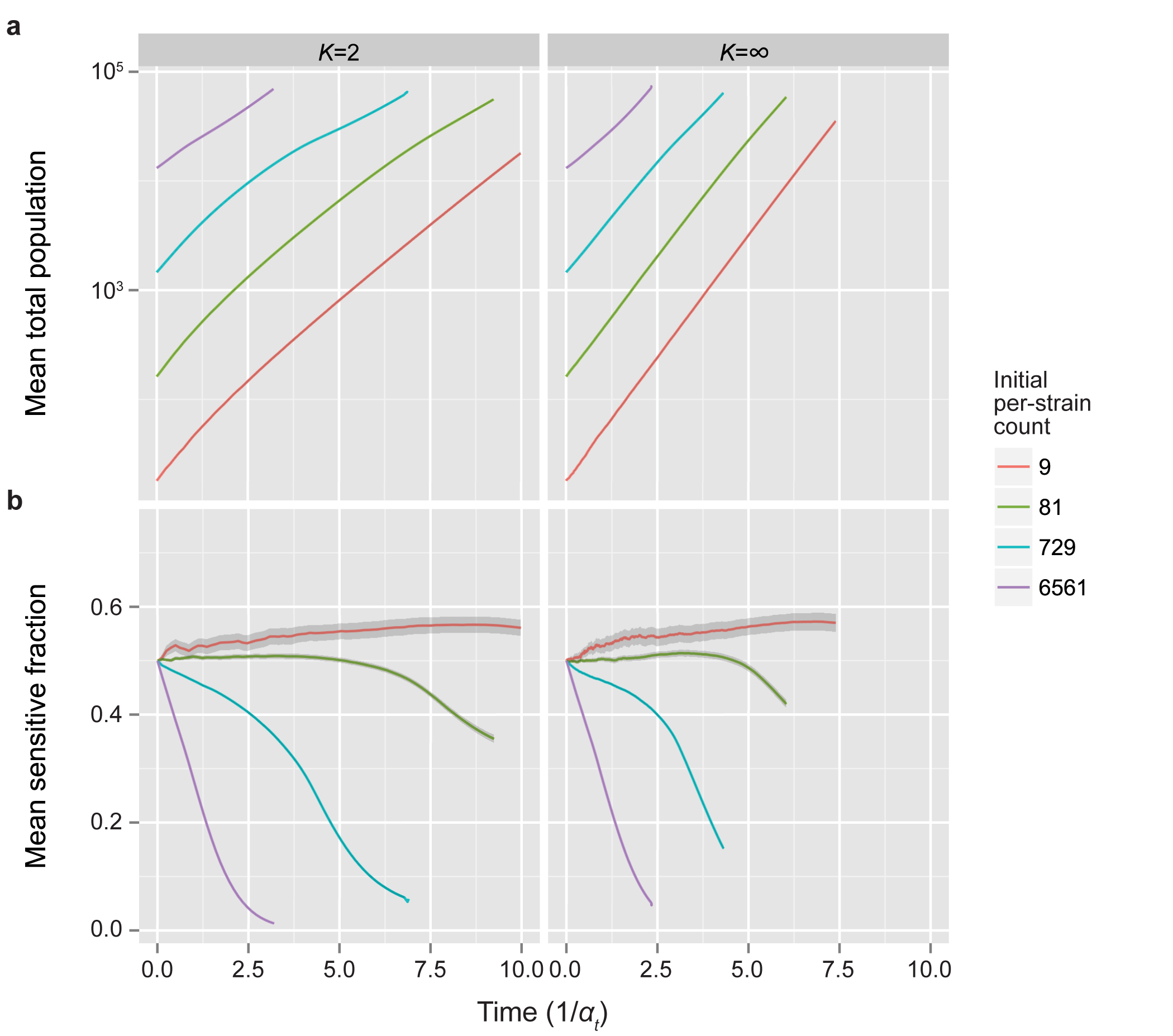

Supplement: S8 Fig — Populations begin with an equal number of T6S+ and sensitive individuals at a specified per-species population, scattered over an r 0 = 84 domain, and grow until the radius has doubled. (a) Population over time for nutrient-limited growth (K = 2, left) and non-limited growth (right). Error ribbons smaller than data curve. (b) Mean sensitive fraction over time for nutrient-limited growth (K = 2, left) and non-limited growth (right). For both panels, n = 40 per condition; scaling factor λ = 100. Ribbons = 1 S.E. (TIF) [file pcbi.1004520.s017.tif]

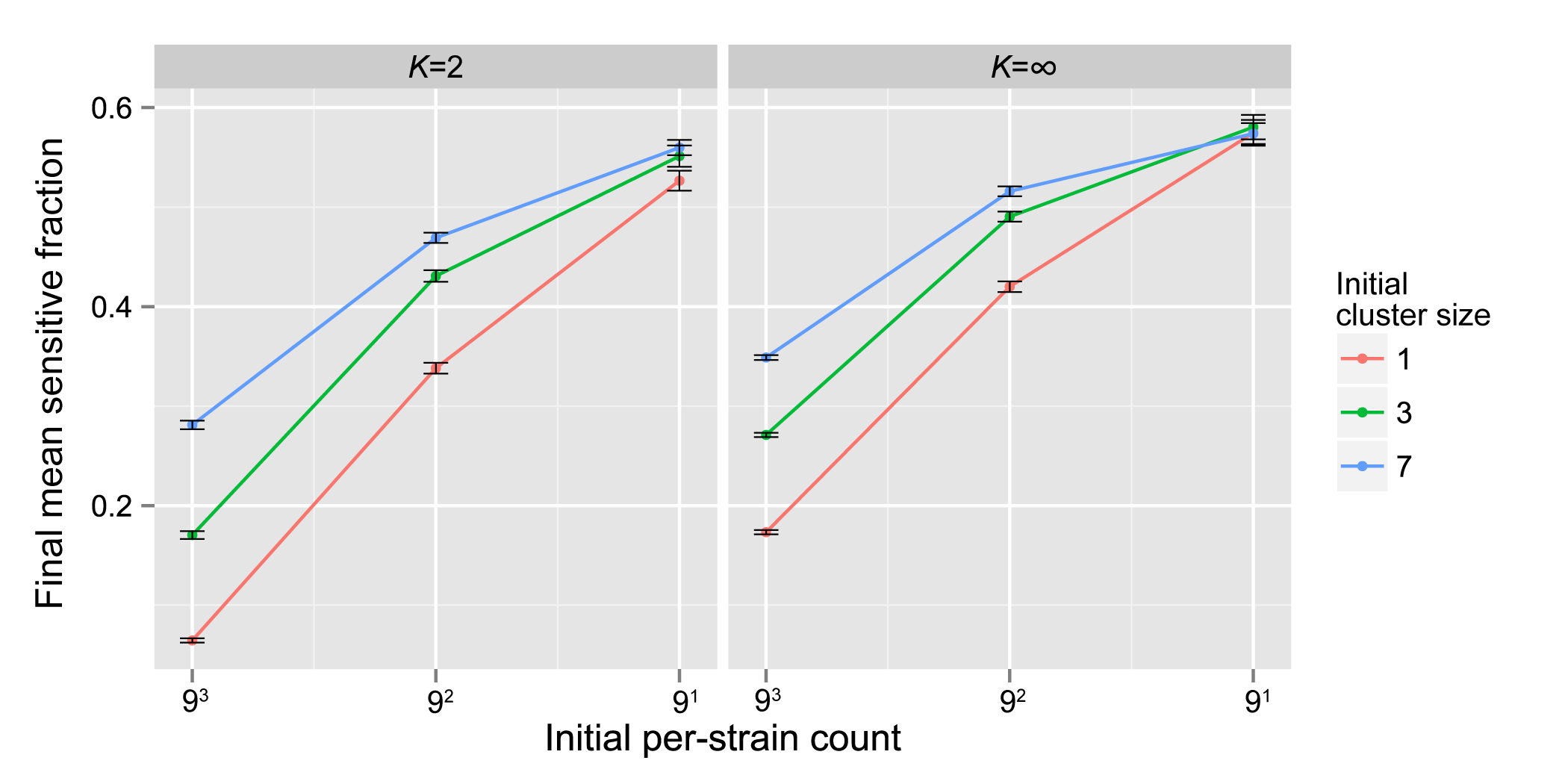

Supplement: S9 Fig — Initial populations are placed in compact groups of m = 1, 3, or 7 individuals, and with strict separation between these clusters. Shown is final sensitive fraction as a function of initial per-species count. Populations begin with a specified per-species population, scattered over an r 0 = 84 domain, and grow until the radius has doubled. n = 90 per condition; scaling factor λ = 100. Error bars = 1 S.E. (TIF) [file pcbi.1004520.s018.tif]
