# Supplementary material for: Established Microbial Colonies Can Survive Type VI Secretion Assault
Source: PLoS Comput Biol. 2015 Oct 20;11(10):e1004520. doi: 10.1371/journal.pcbi.1004520 (PMC4619000; doi:10.1371/journal.pcbi.1004520)
Supplement: S1 Table — For range expansion simulations, all cells within a specified Manhattan distance (“Innoculum radius”) are included in the founding population. The resulting population (“Innoculum population”) depends on the lattice geometry. (PDF) [file pcbi.1004520.s019.pdf]

Table S1. Simulation geometries for range expansions.

Lattice	Inoculum population	Inoculum radius
1D (linear)	500	250
2D (triangular)	469	12
3D (cubic)	377	6
