# Supplementary material for: Established Microbial Colonies Can Survive Type VI Secretion Assault
Source: PLoS Comput Biol. 2015 Oct 20;11(10):e1004520. doi: 10.1371/journal.pcbi.1004520 (PMC4619000; doi:10.1371/journal.pcbi.1004520)
Supplement: S2 Table — (PDF) [file pcbi.1004520.s020.pdf]

Table S2. Simulation behavior definitions.

Name	T6S- (sensitive)	T6S+ (self-immune)
`attack`	Do nothing.	With probability γ, trigger the `be-attacked` behavior in one occupied neighbor (if one exists); otherwise, do nothing.
`be-attacked`	Die.	Do nothing.
`reproduce`	With probability α_s, divide into an adjacent space; otherwise do nothing.	With probability α_t, divide into an adjacent space; otherwise do nothing.
