# Supplementary material for: Established Microbial Colonies Can Survive Type VI Secretion Assault
Source: PLoS Comput Biol. 2015 Oct 20;11(10):e1004520. doi: 10.1371/journal.pcbi.1004520 (PMC4619000; doi:10.1371/journal.pcbi.1004520)
Supplement: S3 Table — (PDF) [file pcbi.1004520.s021.pdf]

Table S3. Parameter ranges for comparison of predicted to simulated rates of sensitive strain growth.

	Attack rate ($\tilde{\gamma}$)			Sensitive strain growth rate (α_s)			Inoculum radius (r_0)		
	min	max	step	min	max	step	min	max	step
1D	1	20	1	1	4	1	1	20	1
2D	0	14	2	1	4	1	3	11	2
3D	0	14	2	1	4	1	3	11	2
