# Supplementary material for: Established Microbial Colonies Can Survive Type VI Secretion Assault
Source: PLoS Comput Biol. 2015 Oct 20;11(10):e1004520. doi: 10.1371/journal.pcbi.1004520 (PMC4619000; doi:10.1371/journal.pcbi.1004520)
Supplement: S4 Table — (PDF) [file pcbi.1004520.s022.pdf]

Table S4. Active population growth rates for various division capacities.

Division capacity (K)	Highest root of μ	Dominant eigenvalue of $\hat{L}$
2	0.618	0.618
3	0.839	0.839
4	0.928	0.928
5	0.966	0.966
